# Multitarget brain implants enable generalized decoding of Parkinson’s disease symptoms from chronic home recordings

**DOI:** 10.21203/rs.3.rs-9125364/v1

**Published:** 2026-05-15

**Authors:** Timon Merk, Maria Olaru, Nanditha Rajamani, Patricia Zvarova, Erblina Purelku, Zixiao Yin, Richard M. Köhler, Jojo Vanhoecke, Ashwini Oswal, Guanyu Zhu, Jian- Guo Zhang, Reza Abbasi-Asl, Amelia Hahn, Simon Little, Andreas Horn, Philip A. Starr, Wolf-Julian Neumann

**Affiliations:** 1 Movement Disorder and Neuromodulation Unit, Department of Neurology, Charité – Universitätsmedizin Berlin, Berlin, Germany; 2 Department of Neurosurgery, Baylor College of Medicine, Houston, TX, USA; 3 Neuroengineering Initiative, Rice University; 4 Department of Neurological Surgery, University of California San Francisco, San Francisco, United States of America; 5 Institute for Network Stimulation, Department of Stereotactic and Functional Neurosurgery, University Hospital Cologne, Germany; 6 Department of Neurosurgery, Beijing Tiantan Hospital, Capital Medical University, Beijing, China; 7 Beijing Key Laboratory of Neurostimulation, Beijing, China; 8 MRC Brain Networks Dynamics Unit, Nuffield Department of Clinical Neurosciences, University of Oxford, Oxford, UK; 9 Department of Bioengineering and Therapeutic Sciences, University of California San Francisco, San Francisco, United States of America; 10 Department of Neurology, University of California San Francisco, San Francisco, United States of America; 11 Einstein Center for Neurosciences Berlin, Charité – Universitätsmedizin Berlin, Berlin, Germany

## Abstract

Adaptive deep brain stimulation (aDBS) oèrs unprecedented precision in the treatment of Parkinson’s disease. Current aDBS control algorithms depend on brain signal biomarkers, such as basal ganglia oscillatory activity in the beta (8–30 Hz) range. Even after extensive optimization through a specialized medical team, about one third of patients may remain ineligible for aDBS due to insuìcient biomarker fidelity. Moreover, while the marker broadly correlates with disease severity, it does not recognize how specific symptoms independently fluctuate over time. A concept to address these shortcomings is to rely on machine learning based brain signal decoding. Here, we trained decoders on over 500 hours of invasive multisite recordings from cortex and deep brain targets to predict the wearable based estimates of PD symptoms and side-eècts without patient individual training. Recordings were streamed from brain implants while patients were at home on their usual antiparkinsonian treatment. The resulting models robustly outperformed individually defined beta activity, while providing three major conceptual advances: they performed even in patients without a basal ganglia beta rhythm, generalized across patients without requiring retraining or adaptation and robustly and dièrentially and simultaneously decoded the severity of bradykinesia, tremor and dyskinesia. In a proof-of-concept simulation, we demonstrate how these models could be used to steer stimulation fields dynamically to predefined symptomresponse brain networks. This fusion between adaptive and connectomic DBS may define when to stimulate which brain circuit for optimal symptom-specific control in real time and pave the way for a new generation of fully automatized symptom-specific neuromodulation approaches.

Parkinson’s disease (PD) is the fastest growing neurodegenerative disease (Schiess et al., 2022). As a neural systems disorder, it is associated with a multitude of motor symptoms, often accompanied by neuropsychiatric comorbidity and medication induced side- eècts. Deep brain stimulation (DBS) is an eèctive treatment for PD patients with motor complications, but identifying the optimal stimulation settings that fits all symptoms is costly and time-consuming for both patients and clinical teams (Lozano et al., 2019). Moreover, such an optimal setting cannot always fully ameliorate the dramatic symptom fluctuations that often constitute one of the key motivations of patients to seek neurosurgical interventions.

A novel stimulation paradigm called adaptive DBS addresses this clinical need by modifying the intensity of stimulation dynamically to symptom and medication states over time (Arlotti et al., 2018; Little et al., 2013; Neumann et al., 2019). In brief, adaptive DBS uses invasively recorded brain signals, most often beta oscillatory activity (8–30 Hz) or gamma (60–90 Hz), as a proxy of the PD symptom state, to dynamically adjust stimulation intensity with high temporal precision (Busch et al., 2024; Little et al., 2013; Oehrn et al., 2024; Stanslaski et al., 2024). The recent FDA approval and CE marking of fully implantable adaptive DBS systems represent a milestone in translating invasive neurotechnologies from the laboratory to clinical practice, enabling rapid, moment-to- moment adjustments of therapy to the changing situations patients with Parkinson’s disease encounter in their daily lives.

However, the current approach of relying on a single oscillatory feedback signal recorded directly from the DBS electrode has the following issues and may not fully realize the potential of this paradigm and presents unresolved challenges.

Three specific barriers have to be overcome to open new horizons for broad clinical adoption of this technology. First, previous studies report that a significant proportion of patients remain ineligible for beta based adaptive DBS due to artifact contamination, low signal amplitude, or uncertaintly about the fidelity with which beta activity tracks symptoms severity in a given individual (Stanslaski et al., 2024). Second, a major concern of aDBS is the additional parameter space that needs to be explored and defined by clinicians who will require significantly more time and in-depth knowledge on invasive neurophysiology and neural engineering, leading to increased treatment costs (Neumann et al., 2023). If the programming process cannot be automated, this will limit aDBS treatment to specialized centers and may hinder translation of DBS treatment to community hospitals and coverage in rural areas. Third, the single feedback signal may permit only a coarse adjustment between the dopaminergic or clinical “Ò” and “On” states of Parkinson’s disease, without the resolution needed to capture the dynamic expression of individual symptoms. As a complicating factor, beta activity and other brain rhythms are modulated by alternate healthy states, such as movement (Köhler et al., 2024; Mathiopoulou et al., 2024; Merk et al., 2022a), sleep (Yin et al., 2023), cognitive control (Zavala et al., 2018), modulation through circadian fluctuations (Cagle et al., 2024; van Rheede et al., 2022) and can even inversely correlate with some symptoms, such as tremor, leaving the patient with suboptimal tremor control (He et al., 2023).

Meanwhile, first attempts for symptom specific treatment of PD were reported through the use of brain network wiring diagrams developed in the field of connectomic DBS (Rajamani et al., 2024). In this work, specific subsets of cortical projection fibers were associated with optimal DBS response in bradykinesia, rigidity, tremor, and axial symptoms such as gait. While this would allow static changes in settings (e.g. to steer current to maximally engage with the tremor circuit for tremor-dominant patients), symptom-constellations change over time. For instance, in stressful moments, resting tremor is known to rise, but may be absent in other times of the day, in which, independently, bradykinetic or dyskinetic symptoms may dominate symptom burden. Advances in connectomic DBS alone do not provide the necessary means to respond to such dynamical symptom fluctuations, which are not consistently present at the same time.

With the present study, we aim to demonstrate how machine learning based brain signal decoding could overcome all of the three abovementioned barriers of aDBS and bridge the gap between adaptive and connectomic DBS, an advance that would combine the optimal targeting of symptom-specific networks in space with dynamic optimization to symptom fluctuations over time.

## Results

### Patient disposition and analytic workflow

**Figure 1 –:**
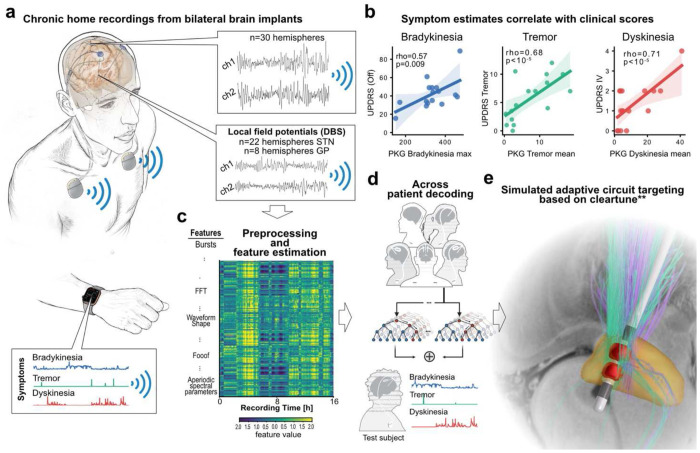
Machine learning based multi-symptom decoding from invasive cortical and subcortical recordings. a) Cortical and subcortical invasive electrophysiological recordings were obtained in addition to wearable symptom estimates (PKG-watch) (Olaru et al., 2024) for 16 Parkinson’s disease patients. Data was continuously recorded while patients were at home in their natural environments. b) Wearable symptom estimates showed for bradykinesia, tremor and dyskinesia significant correlations with groundISP]truth clinician assessed UPDRS (Unified Parkinson’s disease rating scale) scores. c) The py_neuromodulation toolbox (Merk et al., 2023) was used to pre-process neural recordings and estimate different neural features such as bursts, temporal waveform shape, aperiodic spectral parametrization and oscillatory features. d) Bradykinesia, dyskinesia and tremor symptoms were predicted within a leave one subject and hemisphere out cross validation. e) In a simulation study respective circuits could then be stimulated based on the patient-individual and time-resolved symptom predictions.

We analyzed 581 hours (> 24 days) of invasive brain signal data recorded at-home from 16 subjects (14 male, 2 female) with Parkinson’s disease implanted with an investigational bidirectional neural interface, Summit RC+S (Medtronic Inc.), designed for chronic sensing of subcortical and cortical field potentials during activities of daily living (Stanslaski et al., 2018) (Figure 1a, Supplementary Figure 1). DBS leads were implanted either targeting the subthalamic nucleus (11 participants; Medtronic 3389 lead) or globus pallidus (five participants; Medtronic 3387 lead) and four-contact electrocorticography (ECoG) strips were placed subdurally over precentral and postcentral gyri. Sixteen participants with PD were recruited for a parent study of chronic multi-site brain recording, using the Summit RC+S investigational device, to understand the physiological basis for Parkinsonian motor signs and implement adaptive DBS (ClinicalTrials.gov registration: NCT03582891). Data from this cohort have been reported in prior publications with distinct objectives (Gilron et al., 2021; Oehrn et al., 2024; Olaru et al., 2024). Participants recorded neural data in their home environments while wearing Personal KinetiGraph (PKG, Global Kinetics Pty Ltd.) monitors (Griìths et al., 2012) on both wrists during routine daily activities and while on their usual preoperative antiparkinsonian medication regimen (Table 1).

The PKG is a wrist-worn device equipped with a three-axis accelerometer and a commercially validated algorithm that continuously estimates tremor, bradykinesia, and dyskinesia every two minutes (Horne et al., 2015). To verify that the wearable output reflects clinically meaningful symptom estimates in this cohort, we correlated each symptom score with pre-operative clinical symptom severity obtained by specialized neurologists through the Unified Parkinson’s Disease Rating scale (Figure 1b). We found significant correlations for each symptom (bradykinesia: r=0.57, perm. test p=0.009; tremor: perm. test r=0.68, p<0.001; dyskinesia: r=0.71, perm. test p<0.001) confirming previous validation reports (Braybrook et al., 2016; Griìths et al., 2012; Horne et al., 2015).

### Brain signal decoding outperforms beta activity in the prediction of PD symptoms

**Figure 2 –:**
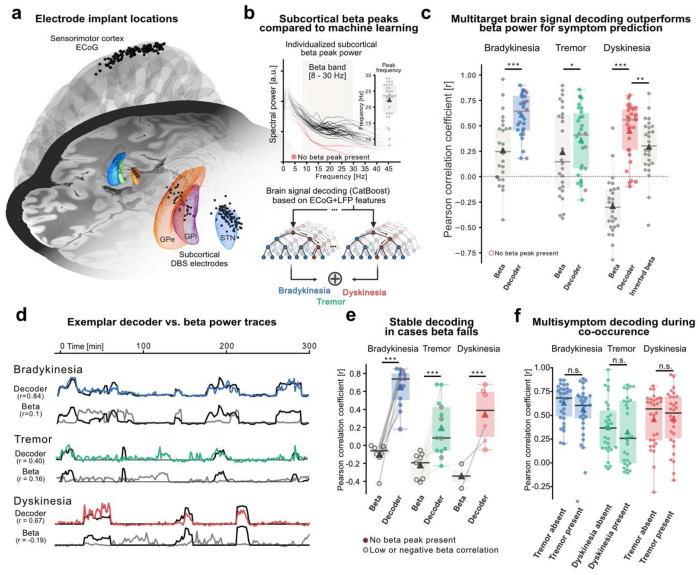
Machine learning based decoding outperforms beta oscillation based symptom predictions. a) Individual cortical and subcortical recording contacts. b) all subcortical signals were visually inspected for beta peaks within the range (shaded area) defined in current adaptive DBS implants (Stanslaski et al., 2024). Peak frequencies No beta peaks were observed in four hemispheres (red lines). Individual beta peak correlations were compared to decoder output from CatBoost models for each of the three symptoms provided by the wearable, Bradykinesia, Tremor, Dyskinesia. c) Multitarget and multifeatured brain signal decoder significantly outperform subcortical beta correlations across all symptoms and provide output in cases with no beta peak. For Dyskinesia the inverted peak-signal correlations are additionally shown, because dyskinesia can be negatively correlated with beta activity. d) Exemplar decoder vs. beta traces for a 300-minute recording window for each symptom in comparison to the true wearable output. e) In some cases a beta peak was not present (red circles) and in some cases beta was not correlated with symptoms. Decoder output provided stable symptom estimations across these cases despite failure of beta activity. f) Comparison of decoder output performs during absence and presence of additional symptoms reveals stable decoding performance with minimal symptom interference (all p>0.05).

We benchmarked our machine learning approach against beta-based adaptive DBS in PD that is considered clinical state-of-the-art and has recently received FDA-approved and CE-certification (Bronte-Stewart et al., 2025; Stanslaski et al., 2024). To this end, we compared our models in their ability to predict bradykinesia, tremor and dyskinesia estimates with individually optimized spectral beta power recorded from the DBS electrode. Following clinical practice, patient-individual beta peaks were identified from subcortical power spectra within the 8–30 Hz frequency range, with 5 Hz spectral windows surrounding the peak. Distinct subcortical beta peaks were present in 21/24 STN and 5/6 GP recordings (26/30 hemispheres). The average peak frequency was 22.2 ± 5.77 Hz across all nuclei (STN: 22.14 ± 5.69 Hz; GP: 22.5 ± 6.65 Hz; Figure 2a). Notably, despite the commercial availability of sensing enabled brain implants that allow for chronic subcortical recordings, correlations between beta activity and and continuous symptom assessments over many medication cycles have so far only been shown in a handful of subjects (Gilron et al., 2021). Here, we report cohort results for within subject correlations of beta activity with synchronized long-term symptom assessments using PKG symptom estimates that were captured every 2 minutes (restricted to daytime – 8 am to 8 pm). We observed positive correlations for beta activity with bradykinesia (r=0.26 ± 0.32, perm. test p<10^−5) and tremor (r=0.24 ± 0.42, p=0.007) and negative correlations for dyskinesia (r=-0.29 ± 0.27, perm. test p<10^−5) (Figure 2b). These results corroborate the potential utility of beta activity as a biomarker for adaptive DBS across long postoperative recording spans.

Notwithstanding this, we hypothesized that machine learning based approaches can significantly outperform the precision of beta-based symptom predictions while also providing means to overcome the barriers of signal fidelity requirements, timeconsuming parametrization and lack of symptom specificity. Therefore, we trained brain signal decoders on multisite recordings to directly predict symptom scores provided by the wearable as the decoding target variables (Merk et al., 2023). To this end, we instantiated a virtual signal stream in our fully online compatible brain signal decoding platform *py_neuromodulation* for all cortical and subcortical electrodes from each hemisphere, through which all brain data were processed in synchrony to the 2-min wearable output, including filtering, artifact correction and spectral and temporal feature estimation (Figure 1c). Features entailed oscillatory rhythms, temporal waveform shape, oscillatory burst dynamics and aperiodic spectral parametrization (Cole and Voytek, 2017; Donoghue et al., 2020; Tinkhauser et al., 2018). Given its simplicity and availability in neurotechnology, we further added the time of day (clock) as a system-level feature.

Next, we compared the performances of dièrent machine learning methods (CatBoost, Random Forest, XGBoost, PCA + Linear Model, Linear Model) for predicting symptom scores with leave-one-hemisphere out (LOHO) and leave-one-subject out (LOSO) crossvalidation. This revealed that CatBoost consistently achieved the highest decoding performances across symptoms (see Supplementary Fig. 2). CatBoost is a gradient boosting algorithm belonging to a class of machine learning methods that has previously been shown to outperform alternative approaches in brain signal decoding (Hirschmann et al., 2022; Merk et al., 2022b; Yao et al., 2020). Gradient boosting models construct ensembles of shallow decision trees sequentially, with each tree trained to correct the residual errors of its predecessors. This learning paradigm mitigates data leakage and overfitting and yields predictions that generalize robustly across heterogeneous patient cohorts. On this methodological basis, CatBoost was selected for all subsequent brain signal decoding analyses. Cross-validation and test-set performance were pragmatically evaluated using a leave-one-hemisphere-out scheme across subjects, as bilateral recordings were not available for all individuals. Using hemispheres as the unit of holdout increased the number of validation folds while ensuring separation between training and evaluation recordings. Control analyses using leave-one-subject-out crossvalidation are also reported and not yield significantly lower decoding accuracy.

The resulting brain signal decoders significantly outperformed the predictive performance of beta activity (see table 2) in all symptom domains, including bradykinesia (r=0.61 ± 0.19, p<10^−5, Δr_ML-beta_= 0.35 ± 0.42; perm. test p=0.0002), tremor (r=0.39 ± 0.32, perm. test p<10^−5, Δr_ML-beta_= 0.15 ± 0.33; perm. test p=0.029) and dyskinesia (r=0.45 ± 0.27, perm. test p<10^−5, Δr_ML-beta_= 0.16 ± 0.28; perm. test p<10^−5). The performances remained stable when including night-time data (Supplementary Figure 3a) and switching to leave-one-subject-out instead of leave-one-hemisphere-out crossvalidation (Supplementary Figure 3b).

Thus, the multitarget decoders provided more accurate measures of PD symptoms while addressing all three major aDBS challenges outlined above: Regarding signal fidelity, we empirically reproduced that beta activity as a feedback signal was not always recordable. In the present cohort, distinct beta peaks were only present in 86.7% and 26/30 of recordings, in line with previous publications (Darcy et al., 2022; Lofredi et al., 2023; Stanslaski et al., 2024). While beta based aDBS would fail entirely with these recordings, our decoders provided stable performance (n=4 hemispheres; bradykinesia (r=0.6 ± 0.25), tremor (r=0.17 ± 0.21) and dyskinesia (r=0.49 ± 0.29). In eight additional recordings, no significant relationship (r≤0) between beta and the traditional target symptom – bradykinesia – was observed (r=-0.1 ± 0.13 for beta ~ bradykinesia), while multitarget decoders provided significant predictions on all subjects (Bradykinesia neural multi-site decoding for subjects where beta correlation was absent: r=0.67 ± 0.18, perm. test p<10^−5; DBS electrodes only: 0.53 ± 0.19, , perm. test p<10^−5), regardless of whether a subcortical beta peak was present or not (see Table 2). Thus, in this study, beta-based aDBS would have left 40% of patients ineligible for adaptive DBS, while our decoder based approach would have provided robust feedback across all recording sites.

To address the second major challenge, the additional programming burden, we specifically trained our models across subjects, for out-of-the-box utilization. Traditional beta-based systems require patient-specific spectral peak identification and careful threshold tuning, often necessitating time-intensive sessions with specialized clinicians. In contrast, our decoders generated accurate symptom estimates without requiring individualized calibration. This shift reduces dependence on expert programming and iterative parameter adjustments, thereby decreasing the need for repeated in-person visits. Beyond improving patient quality of life through fewer clinic encounters, this approach has the potential to substantially lower healthcare costs by alleviating reliance on scarce, highly specialized neurostimulation expertise, particularly in community and resource-limited care settings. By eliminating the requirement for manual customization, our approach lowers the barrier to widespread deployment of closed- loop neuromodulation and paves the way for more equitable access to precision therapy. Finally, the patient agnostic approach also facilitates more objective and blinded clinical trial designs, enabling the use of standardized models as published with this article across sites without the variability, subjectivity, and potential error introduced by individualized parameter tuning.

The final point addresses the symptom specificity, which is an inherent strength of the symptom specific decoder approach. While beta based adaptive DBS only provides a single measure of symptom severity, we report that dièrential decoding of multiple symptoms provided stable predictions without significant deterioration. Despite the potential movement related impact of tremor, decoding of bradykinesia (r=0.56 ± 0.23) was stable during episodes of tremor with no significant deterioration when tested against episodes without tremor (both p=0.15). The decoders could even distinguish the nature of involuntary movements, with stable dyskinesia decoding during tremor (r=0.46 ± 0.29, perm. test p=0.95 when tested against episodes without tremor) and stable tremor decoding during dyskinesia (r=0.32 ± 0.34, perm. test p=0.29 when tested against episodes without dyskinesia).

In conclusion, our open-source multitarget machine learning models outperformed beta activity, provided robust feedback even in the 40% (12/30) of recordings that would be ineligible for beta based aDBS and provided estimates of multiple symptoms without requiring any patient specific input.

### Symptom specific feature sets for brain signal decoders

**Figure 3 –:**
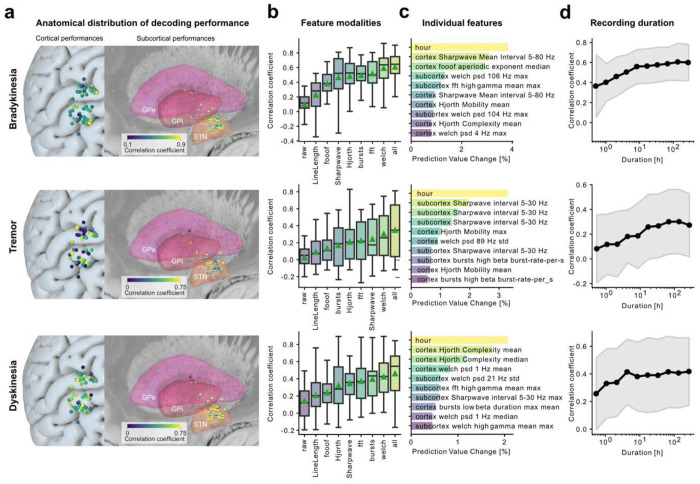
Neural decoding location, machine learning architecture and feature modality investigation. a) Recording contacts were color-coded for channel-individual decoding performances for cortical and subcortical recordings. b) Decoding performances for different feature modalities were computed. Best modalities were for each symptom *all* computed feature modalities. c) Most important CatBoost-model features were estimated using the Predictive Value Change metric. The time of day “hour” feature showed highest importance for each symptom decoding. Performances stayed however comparable excluding the time feature (Supplementary Figure 3d). d) Learning curves were computed by randomly selecting a subset of samples. A plateau of performance could be observed for after 10 h of training data, but performances would have likely been improved with further training data.

To investigate the importance of surgical target for decoding performances, we investigated channel-individual decoding for the dièrent subcortical nuclei (Figure 3a). For bradykinesia and dyskinesia we found that STN performances were significantly higher compared to GP (correlation coèicient bradykinesia: STN r=0.50 ± 0.20, GP r=0.23 ± 0.35, perm. test p<0.001; dyskinesia: STN 0.48 ± 0.22, GP 0.22 ± 0.28, perm. test p<0.001) (Supplementary Figure 3c). For tremor, GP performances were higher than STN (regression correlation coèicient tremor STN 0.25 ± 0.31, GP 0.36 ± 0.33, perm. test p=0.054). The additional use of ECoG signals however, led to robust performance across all symptom domains with no significant dièrences between STN and GP DBS targets (all p>0.05).

We next aimed to get further insight into the most informative features for decoding bradykinesia, tremor and dyskinesia with CatBoost across patients. To this end, we dièrentially tested separate “feature modalities” such as waveform shape, fast fourier transform and more provided by the *py_neuromodulation* pipeline (Merk et al., 2023). We found that for all decoded symptoms, the combination of all features resulted in best performances (Figure 3b). To identify particularly performative individual features, we objectified the feature importances using the “predictive value change” method, which quantifies how much predictions change on average if individual features were changed. The best electrophysiological features varied by symptom and included oscillatory activity, waveform-shape and Hjorth features (Figure 3c). Notably, neither beta `t nor beta burst features were strong contributors to the decoding performances.

Overall, the best feature for every symptom decoder was the “hour” of the day feature, thus we quantified to what degree the entire approach depended on it. After removal of the clock feature we found that decoding remained stable across all symptoms without significant drops in performance (bradykinesia r=0.57 ± 0.28 perm. test p=0.51; tremor: r=0.30 ± 0.33 perm. test p=0.06, dyskinesia: 0.45 ± 0.28 perm. test p=0.72; Supplementary Figure 3d).

Finally, we examined the influence of training data availability by relating decoding performance to the cumulative duration of streamed multisite recordings. For each symptom, performance increased sharply with additional data, approaching a plateau at approximately ten hours of recording.(Figure 3d). For Dyskinesia and Tremor, the performance would likely further improve through additional training data. Furthermore, adding more data could make it viable to train more complex model architectures that weren’t tested in this study.

### Brain signal decoding enables adaptive circuit targeting DBS

**Figure 4:**
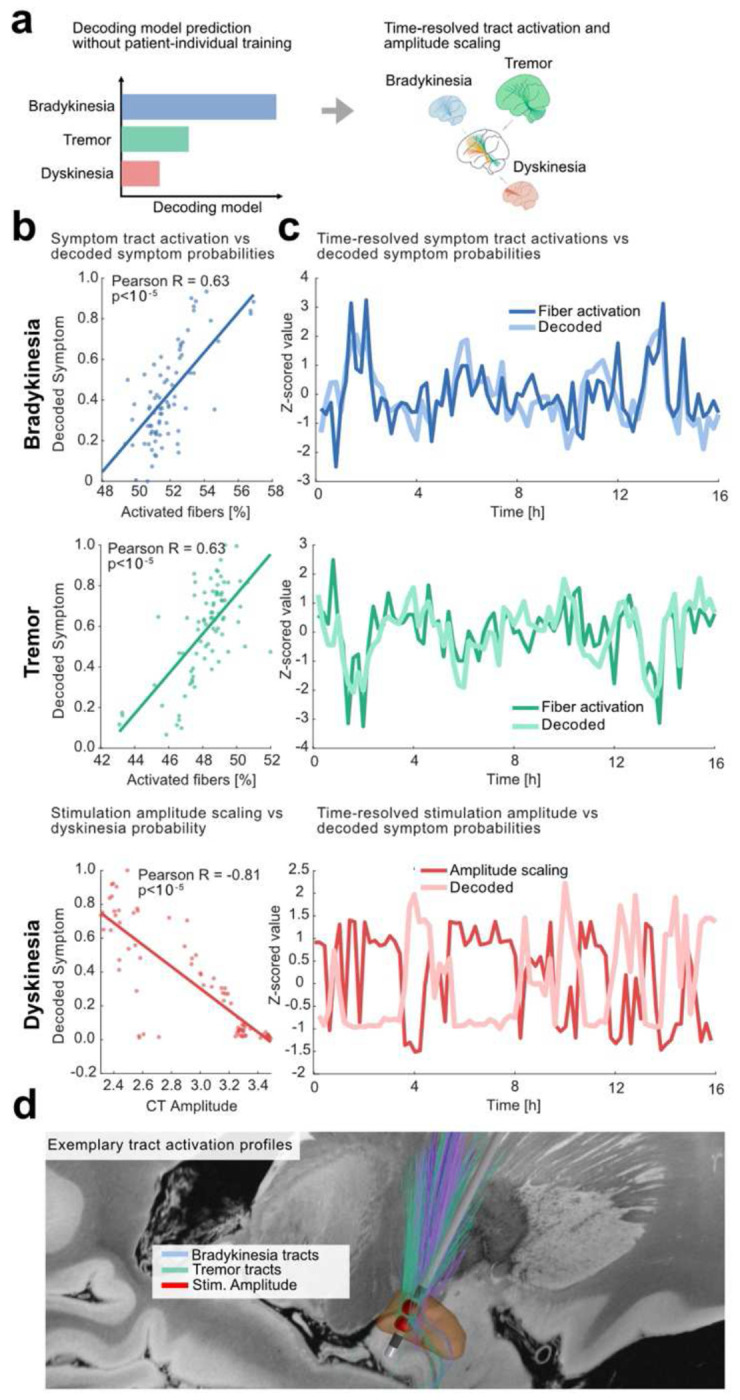
Activation of different Parkinsonian symptom tracts using neural symptom decoding. a) Schematic illustration of stimulation parameter optimization using *cleartune* (Rajamani et al., 2024) according to neural decoding model predictions. Bradykinesia and Tremor tracts were calculated by Rajamani et al. previously. Dyskinesia predictions were applied to inversely scale stimulation amplitude. b) Percentage of optimized tracts showed significant correlations with neural decoding symptom classification correlations for Bradykinesia (top), Tremor (middle) and Dyskinesia (bottom) with stimulation amplitude. c) Time-resolved visualization of decoded symptoms, activated tracts, and stimulation amplitude. d) Exemplary tract activations are represented for all symptoms. Time-resolved tract activation profiles are visualized in dynamic (video) format, provided by Supplementary Video 1.

Our results demonstrate that machine learning brain signal decoding based on multitarget neurophysiology can overcome significant barriers of single-site beta-based aDBS approaches. An important question that remains, is how these decoders could be implemented in a clinical algorithm that translates into maximizing symptom alleviation and minimizing side-eècts from stimulation. Defining optimal control policies for adaptive DBS is a complex endeavor that again requires significant time and èort from clinicians, even if only one feedback signal is being used. Thus, the current challenge for next-generation closed-loop approaches is to reduce clinician programming time, while improving therapeutic specificity. Here, we demonstrate a proof-of-concept simulation, how the fusion of connectomic and adaptive DBS could achieve this. This concept was recently introduced as adaptive circuit targeting DBS (Horn and Neumann, 2025) and builds on the *cleartune* algorithm that optimizes DBS parameters to adjust stimulation location and amplitude to target optimal symptom response tracts in Parkinson’s disease (Rajamani et al., 2024). In the original study, these symptom-wise sets of fiber tracts were defined and validated across 237 patients from five dièrent clinical centers. An algorithm, *cleartune*, was trained to suggest stimulation settings that would maximize stimulation of a weighted combination of these fiber sets, for instance focusing on modulating tremor circuitry in tremor-dominant patients. While originally conceptualized as an automatized programming tool to define *chronic* stimulation settings, here, we apply *cleartune* dynamically, informed by the temporally fluctuating symptom decoders described above. The brain signal decoders informed the algorithm about the present state of symptoms, allowing *cleartune* to dynamically provide optimal stimulation parameters that adjust stimulation fields to maximize fiber tracts responsive for each of the symptoms present, right at the time a symptom occurs. In a feasibility experiment, we computed these temporally resolved solutions for an example patient (patient 1, table 1). We used decoder based symptom estimates to compute tract-activations for temporal bins of 12 minutes. For bradykinesia and tremor scores we normalized symptom predictions for every time step, and used dyskinesia predictions to scale stimulation amplitude. This design reflects the distinct clinical roles of these signals, with dyskinesia predictions used to directly scale stimulation amplitude in order to prevent overstimulation and reduce stimulation-induced side eècts, while bradykinesia and tremor estimates were normalized to capture dièrential symptom dynamics over time. Notably, the primary aim was to create a fusion of physiology and connectomics in the temporal domain. An important limitation of this first simulation is that the decoder was not àected by actual changes in stimulation settings that are simulated, which could bias and deteriorate decoding accuracy in a real-world scenario. The time-resolved symptom tract activations showed a significant correlation with decoded symptom severities (Pearson correlation bradykinesia r=0.63, p<10^−5; tremor r=0.63, p<10^−5). For dyskinesia the decoded symptom severity showed significant correlations with stimulation amplitude (Pearson correlation r=-0.81, p<10^−5). These results are circular but demonstrate that *cleartune* functioned as intended in such a dynamic setting. Exemplar symptom states and fiber tracts are shown in figure 4, and the dynamic symptom network tract progression is visualized in Supplementary Video 1. In the future, this framework could be extended for other disorders and symptoms of neurological and psychiatric disorders (Hollunder et al., 2024, 2021), stimulating the right network at the right time. Moreover, it could directly be used to guide the avoidance of connections associated with side-eècts (such as dyskinesia or nonmotor symptoms (Irmen et al., 2020; Meyer et al., 2025), in the future, as well.

## Discussion

This study demonstrates that machine learning-based brain signal decoding from multisite cortical and subcortical recordings substantially outperforms basal ganglia beta activity, currently in widespread use for clinical adaptive deep brain stimulation (aDBS), across multiple important motor symptoms of Parkinson’s disease. Prior studies (Gilron et al., 2021; Olaru et al., 2024) showed in this same data set that decoding of motor state (on/ò states in Gilron et al (Gilron et al., 2021), dyskinesia in Olaru et al (Olaru et al., 2024)) could be accomplished in naturalistic home recordings using single frequency bands or linear combinations of frequency bands. Here, in the same data set, we add: 1) a more general, *machine learning based* decoding strategy that combines frequency domain and time domain metrics, 2) a cross-subjects decoding strategy that avoids having to build patient-specific models, and 3) evaluation of simultaneous multisymptom decoding. Moreover, we show a proof-of-principle analysis of how these decoded states could be used to inform circuit-aware adaptive DBS programming by steering the stimulation field toward the symptom-response tracts in a given patient at the right time when symptoms appear (Horn and Neumann, 2025). In this cohort of 16 patients monitored over 581 hours of naturalistic home recordings, symptom-specific decoders achieved markedly higher predictive accuracy than subcortical beta power, increasing predictive accuracy by approximately 135% for bradykinesia (r = 0.61 ± 0.19), 63% for tremor (r = 0.39 ± 0.32), and 55% for dyskinesia (r = 0.45 ± 0.27), relative to betabased correlations. Critically, the decoder framework addressed key limitations of betabased aDBS. First, it overcame the fidelity barrier. In 40 percent of recorded hemispheres, beta activity was absent or non-informative, yet decoding performance remained robust. Second, it removed the need for patient-specific spectral peak identification, enabling out-of-the-box applicability without manual programming. Third, it enabled simultaneous and distinct tracking of multiple symptoms, a prerequisite for personalized precision neuromodulation. In a simulated integration with connectomics informed algorithmic control policies, this decoding approach could dynamically steer stimulation to symptom-optimized fiber tracts in real time, as recently outlined (Horn and Neumann, 2025). Together, these advances the conceptual and technical framework for neural mulittarget/multisignal decoding for scalable personalized closed loop neuromodulation for Parkinson’s disease.

Moreover, our study provides important insights for the advancement of adaptive DBS. Despite the fact that beta activity was primarily chosen as a state-of-the-art benchmark for decoder precision, we provide robust cohort evidence for the long-term relationship of beta activity with synchronized symptom estimates of bradykinesia and tremor. Prior chronic studies have typically yielded temporally sparse in-clinic observations (Stam et al., 2025) limiting insight into the dynamic evolution of neural biomarkers across real-world contexts and extended timescales. Our study shows that on a cohort-level, there is a robust but moderate correlation between beta activity and synchronized estimates of bradykinesia, tremor and dyskinesia.

The demonstration of better correlations of our multitarget decoding framework despite the more conservative cross-validation across subjects highlights the potential of machine learning applications in closed-loop neuromodulation. We further emphasize that the developed decoders can be applied to neural signals without requiring a prolonged temporal window. A two-minute recording segment and time-of-day is sufficient to generate predictions of the here investigated symptoms.

The pre-trained models that we release together with this publication can be readily applied for out-of-the-box use and validation in prospective trials. If successful, building neurotechnologies based on such open models could significantly reduce healthcare costs at the societal level, by improving treatment access for patients previously ineligible (e.g., 40% for beta-based aDBS in the present cohort), reducing need for clinical visits and programming burden and allowing for refined symptom specific treatment optimization without patient individual parametrization.

Importantly, our study is not the only one presenting evidence for decoder-based symptom detection from brain signals. Hirschmann et al. showed that tremor could be classified in an acute externalized setting within the hospital using LFP signals recorded from the STN (Hirschmann et al., 2017). Lauro et al. presented bradykinesia and tremor decoding within an intraoperative motor task using micro-, macro- and ECoG-recordings (Lauro et al., 2023). However, our work extends these previous studies in multiple domains. First, we utilized chronic “at home” recordings during naturalistic behavior on regular patient medication that was not bound to specific task recordings or lab settings. Moreover, we showcase robust differential decoding across three of the most important symptoms, namely bradykinesia, tremor and dyskinesia in a cohort of 16 patients implanted in the two most important DBS targets, the globus pallidus and subthalamic nucleus. These symptoms were decoded simultaneously by the same unified models. Our comparison of machine learning methods highlighted gradient boosting as a promising lightweight approach for on-device decoding designs, that can outperform more complex neural network based algorithms, even when originally trained on over 500 hours of neural data. The analysis of training data duration identifies a steep increase in performance even for this relatively simple algorithm until the 10h mark per patient, providing a tangible reference for clinical data science teams in the planning of technology validations. Our feature analysis corroborates the importance of the time-of-day or clock as a powerful feature for symptom decoding (Fleming et al., 2022).

Our study has several important limitations that need to be addressed in future research. Compared to current clinical devices, our approach builds on the additional implantation of electrocorticography strips, which currently do not have regulatory approval for chronic clinical treatment and do not yet provide clinical benefit for DBS patients, while leading to a theoretical added risk of brain hemorrhage and infection. However, retrospective safety assessments in over 500 patients across multiple centers deemed these risks as minimal and the outstanding signal to noise ratio among other significant benefits have recently led to a call for innovation in this field (Herron et al., 2025). Our study further corroborates the evidence that ECoG signals outperform local field potential recordings for machine learning applications (Herron et al., 2024; Merk et al., 2021). Another limitation is the fact only three of many motor and non-motor signs of Parkinson’s disease have been decoded in our study. While bradykinesia, dyskinesia and tremor are certainly among the most important symptoms, our future work will aim to add gait impairment, sleep stages, speech deterioration and mood as important symptom domains to future versions of our decoding models. For these symptoms, previous work has already shown promising avenues for neuromodulation refinement, including sleep stage aware (Smyth et al., 2023) and gait specific adaptive DBS (Fekri Azgomi et al., 2025; Isaias et al., 2024; Scafa et al., 2025; Wilkins et al., 2024). Moreover, recent brain signal decoding work has demonstrated the ability to track mood in patients with depression (Alagapan et al., 2023; Merk et al., 2023) and first biomarkers for mood in PD specifically have been reported (Johnson et al., 2025). Notably, our study made use of powerful compute clusters to train and validate the machine learning models. For clinical adoption the technical feasibility for on-chip decoding must be demonstrated. However, we believe that this is feasible already, as first specialized hardware designs have demonstrated high decoding performance using similar model architectures (Dixon et al., 2025; Shin et al., 2022; Zhu et al., 2021). In addition, we note that the data used to train and validate the neural decoders were all recorded in the stimulation-off condition. Future neural decoding models should incorporate stimulation parameters and be trained on datasets that include multiple stimulation states.

Beyond the direct application of decoders for adaptive DBS, we would like to highlight that symptom decoding alone can have significant clinical utility for objective monitoring of clinical states, providing important information for treatment decisions. However, to directly improve the quality of lives of patients as part of a closed-loop neuromodulation paradigm, an algorithmic control policy must be designed that may leverage this rich symptom information. With the present study, we provide a clear pathway to clinical implementation for connectomics informed adaptive circuit targeting in real-time, a previously undescribed fusion of connectomic and adaptive DBS. In a novel in silico feasibility analysis, we adapted the previously described *cleartune* (Rajamani et al., 2024) algorithm that utilizes symptom-response-tract information for symptom specific stimulation field optimization based on real patient data. Previously, this algorithm optimized long term stimulation parameters to target symptom specific networks for chronic deep brain stimulation. The present adaptation allows for dynamic adjustments of stimulation parameters to steer stimulation fields to optimal symptom response networks in real time, based on the simultaneous information of multiple symptom decoders. Thus, if the decoder indicates high levels of bradykinesia and absence of tremor, the algorithm targets fiber tracts associated with optimal bradykinesia response. Just when tremor emerges, the decoder will inform the control policy of this change, and the stimulation field is extended to fiber tracts associated with optimal tremor response, which are typically located more posterior on the motor section of the basal ganglia nuclei. Notably, given that both the symptom decoders and the *cleartune* algorithm have been shown performance in out-of-sample data, the combined approach could be fully automated with only minimal input from clinicians to ensure safety and efficacy (Horn and Neumann, 2025). Before this can be implemented at scale, validation in prospective studies is required using novel devices that can not only adapt stimulation amplitude but also steer the stimulation field in space.

To facilitate this, we publish the pretrained models based on the unique invasive brain data across all patients with the required feature estimation code (https://github.com/neuromodulation/PD-neural-symptom-decoding-inference). We, thereby, hope to accelerate the application of neural symptom decoding for closed loop neuromodulation. We believe that our published models could serve transfer learning paradigms. Adding more data in the future could enable the use of more complex model architectures, such as deep neural networks trained through representational contrastive learning (Mathis et al., 2024; Schneider et al., 2023).

In conclusion, our study establishes a foundation for symptom-aware closed-loop neuromodulation in Parkinson’s disease by combining robust, multitarget brain signal decoding with connectomics-informed algorithmic control. Through the integration of large-scale naturalistic recordings, lightweight decoding models, and real-time pathway targeting, we demonstrate a clinically feasible approach that addresses key limitations of current adaptive DBS systems. By releasing pretrained models and feature estimation tools, we provide an open framework to accelerate validation, transfer learning, and future development. This work marks an essential step toward precision neuromodulation systems that adapt dynamically to individual symptom states, ultimately aiming to improve quality of life and broaden therapeutic access for patients with Parkinson’s disease.

## Methods

### Data Acquisition

We enrolled 16 participants with Parkinson’s disease who were implanted with Medtronic’s investigational bidirectional neural interface, Summit RC+S, designed for chronic sensing of field potentials during daily activities (Stanslaski et al., 2018). We connected depth leads to either the subthalamic nucleus (STN, 11 participants) or the globus pallidus (GP, five participants), and placed paddle-type leads subdurally over the precentral and postcentral gyri (Gilron et al., 2021). These participants had been part of a previous study that did not explore symptom decoding without patient-specific training (Gilron et al., 2021; Olaru et al., 2024). We recruited these individuals from the movement disorders surgery clinic at the University of California, San Francisco, in line with the Declaration of Helsinki. This study was approved by the Institutional Review Board (IRB) of the University of California, San Francisco, under a physician-sponsored Investigational Device Exemption (IDE) granted by the U.S. Food and Drug Administration (FDA) (protocol G180097). The inclusion criteria included a diagnosis of idiopathic Parkinson’s disease by a movement disorders neurologist and standard clinical indications for DBS in the subthalamic nucleus or globus pallidus (Follett et al., 2010; Okun et al., 2009). We excluded individuals with significant cognitive impairment, as indicated by a Montreal Cognitive Assessment score of 20 or lower, or an untreated mood disorder, as assessed by a neuropsychologist. A movement disorders specialist assessed baseline motor function using the Movement Disorder Society revision of the Unified Parkinson’s Disease Rating Scale part III (UPDRS III) in both OFF- and ON-medication states before implantation. We evaluated the severity of dyskinesia during daily activities using UPDRS IVa 4.1 and assessed whether motor signs were more severe in one hemibody using UPDRS III OFF-medication scores for rigidity, bradykinesia, and tremor in each limb.

### Surgical implantation

We implanted quadripolar cylindrical leads in the subthalamic nucleus (Medtronic 3389 lead) or the globus pallidus (Medtronic 3387 lead), with 1.5 mm or 3 mm intercontact spacing respectively, and quadripolar paddle-type cortical leads with 10 mm intercontact spacing using previously described methods (Gilron et al., 2021; Swann et al., 2017). The surgeon positioned cortical leads along a parasagittal trajectory, ensuring that two or three contacts were anterior to the central sulcus and 2–4 cm from the midline. We confirmed electrode placement intraoperatively using cone beam CT (O-arm^™^ Medtronic, Inc.) (Shahlaie et al., 2011) fused with preoperative MRI. For each implanted hemisphere, we connected brain leads to a Medtronic Summit RC+S interface (model B35300R) in the ipsilateral pectoral area using 60 cm lead extenders (model 37087). We secured the connection between the lead extender and the implantable pulse generator (Summit RC+S) with medical adhesive to minimize ECG signal leakage into neural recordings (Hammer et al., 2022). Electrodes were configured into two bipolar recording settings, including overlapping montages mapping channels 1-4 into bipolar recorded channels 1-3 and 2-4, and non-overlapping montages mapping channels into 1-2 and 3-4 recordings. We refined precise anatomic electrode localization post hoc using established image analysis pipelines for deep brain stimulation (Horn et al., 2019) and cortical electrodes (Davis et al., 2021). We coregistered post-implantation high-resolution CT images using a rigid, linear affine transformation and resliced them into a preoperative T1-weighted 3 T MRI (Penny et al., 2011). We verified electrode placement by visual inspection and applied brain shift corrections to refine subcortical anatomy coregistration when necessary (Horn et al., 2019). We localized electrodes to CT artifacts and applied additional surface projection corrections to align cortical leads with the MRI-rendered pial surface (Hermes et al., 2010; Penny et al., 2011). For group analyses, we normalized electrode locations into Montreal Neurological Institute space (Penny et al., 2011) and visualized them on either the cortical surface or a standardized Parkinson’s disease-specific subcortical atlas (Ewert et al., 2018).

### Neural signal recording

We analyzed intracranial sensorimotor cortical and basal ganglia field potentials from participants with chronically implanted cortical paddles and depth leads at home while they engaged in daily activities. Recordings began more than one week post-implantation and prior to initiating therapeutic deep brain stimulation one month after implantation. Participants recorded with different overlapping or non-overlapping montages, using the channel-recording montage that provided the most data duration for signal analysis. They streamed neural data from the Summit RC+S interface to a Microsoft Windows tablet through a custom-made graphical user interface, compliant with US Food and Drug Administration code CFR 820.30 (https://openmind-consortium.github.io) (Gilron et al., 2021). Participants with bilateral implants streamed data simultaneously from both hemispheres. Each RC+S device recorded potentials in the time domain from two channels per cortical paddle at sampling rates of 250, 500, or 1000 Hz, enabling up to 30 hours of continuous recording before requiring a recharge.

### Wearable recordings

Participants recorded neural data at home while wearing Global Kinetics Pty Ltd. Personal KinetiGraph^®^ (PKG^®^) monitors on both wrists during normal daily activities and while on their preoperative dose of antiparkinsonian medication (Table 2). This wearable device, equipped with a three-axis accelerometer, provides continuous tremor, bradykinesia, and dyskinesia scores in 2-minute intervals using a proprietary, validated commercial algorithm (Horne et al., 2015). Each device recorded data for up to nine days before requiring a recharge. We validated the wearable symptom scores by correlating them with the UPDRS-III Total, UPDRS-Tremor, and UPDRS IV-1 scores.

### Signal Processing and Feature Estimation

First, data was preprocessed by defining the sample timestamps for local field potential recordings from the RC+S implant using the open-source toolbox “Analysis-rcs-data” in Matlab R2019b (Sellers et al., 2021). For compression and computational performance, we transformed electrophysiological recordings into Apache Parquet format (Vohra et al 2016). We then used the signal processing and feature estimation toolbox py_neuromodulation to iteratively process the streamed recordings (Merk et al., 2023) using a custom data generator wrapper. We estimated features with a feature sampling rate of 0.1 Hz and a non-overlapping batch size of 10 s. For each patient’s hemisphere we selected a single cortical and subcortical channel, based on highest available data recording duration. Since the data was already recorded in a bipolar, either overlapping or non-overlapping montage, we did not further re-reference data. We resampled data to a common sampling rate of 250 Hz with a forward fill of 4 ms, in case a single sample was missing. If further samples were missing, we discarded the epoch to avoid contribution of artifacts that frequently coincided with streaming data. In addition, we applied notch filtering at the line noise frequency of 60 Hz and the 120 Hz harmonic. We computed oscillatory features within the theta (4 – 8 Hz), alpha (8 – 12 Hz), low beta (13 – 20 Hz), high beta (20 – 35 Hz), low gamma (60 – 80 Hz) and high gamma (90 – 105 Hz) frequency bands using Welch and in addition FFT method. Furthermore, we applied log transform to each band power estimate. We computed spectral aperiodic exponents, offset and knee parameters using the specparam toolbox (Donoghue et al., 2020) (max number of peaks: 3, peak threshold: 2, frequency range: 2 – 40 Hz). Also, we computed Hjorth activity, mobility and complexity (Hjorth, 1970) and bursting features (duration, amplitude, burst rate per second and in-burst state) for low beta, high beta and low gamma frequency bands within a time duration of 30 s and a 75^th^ percentile. We estimated line-length, raw amplitude and temporal waveform shape features (prominence, interval, sharpness within two filter ranges: 5 – 80 Hz and 5 –30 H) for each channel. For waveform shape, we extracted features separately for troughs and peaks (distance between troughs: 10 ms, peaks: 5 ms and reverse), and since this results in a set of peaks and troughs per epoch, we computed the mean for the interval feature, and maximum for prominence and sharpness features. We then applied also the same estimator (mean, max) between troughs and peaks. In addition to neural features, we extracted the time of the day “hour” feature. Since wearable PKG values were collected within a time range of 2 min, we computed the mean, median, maximum and standard deviation of each feature modality within the 2 min epoch for each feature. We further averaged PKG wearable labels within a time range of 10 minutes around the current wearable sample for smoothing purposes. An exemplary wearable time series and corresponding computed feature heatmap is displayed in Figure 1b.

### Beta-based symptom decoding

We established a baseline comparison performance with the subcortical beta LFP biomarker. Here we computed beta power based on the Welch Power Spectral Density (PSD) in different frequency bands: low beta (12 – 20 Hz), high beta (20 – 30 Hz), all beta (8 – 30 Hz) and patient-individual beta peaks. Beta peaks were identified either in a 30 minute daytime recording at the beginning of the recording, or using the whole duration time frequency plots. We computed then the Pearson correlation coefficient of each beta power estimate with Bradykinesia wearable PKG values.

### Neural decoding

We performed neural decoding as classification and regression problems separately. We classified bradykinesia when the wearable raw value exceeding 50, and classified tremor when the raw value exceeded 1. For dyskinesia, we set the classification threshold to 0.02 after patient specific maximum-normalization. The symptom specific PKG value histograms are shown in Figure 1b. For the regression decoding problem, we directly predicted the 15 min smoothed PKG wearable scores. To investigate model generalizability, we tested different cross-validation strategies. First, we performed leave-one-hemisphere out cross-validation (Figure 1). We investigated the effect of including only cortical, subcortical, or a combination of cortical and subcortical channel features. Furthermore, we validated those results when both hemispheres of a patient were left out in a “leave one patient out cross validation”. We compared those results to channelindividual decoding with non-shffled three-fold cross validation. As a default model we used CatBoost (default parameters version 1.2.7; Classification / Regression: iterations=500, tree-depth=6, l2_leaf_reg=3.0, feature binarization border_count=254, classification loss function: logloss, balanced class weights, regression loss function: root mean squared error) (Dorogush et al., 2018). To narrow down optimal feature modalities, machine learning models, and optimal training duration, we estimated bradykinesia within a regression problem, and dyskinesia and tremor as classification problems. We tested different machine learning methods: xgboost (default regression and classification parameters version 2.1.2; learning_rate: 0.3, max_depth: 6, sampling_method: uniform, L2 lambda regulation: 1) (Chen and Guestrin, 2016), Random forests using scikit-learn (Pedregosa et al., 2011) (default parameters version 1.5.1; n_estimators: 100, min_samples_split: 2, min_samples_leaf: 1, max_depth: None, max_leaf_nodes: None), Linear models – Logistic regression and linear regression (scikit-learn default parameters version 1.5.1; linear regression: ordinary least squares linear regression; logistic regression with max_iter: 100, solver: lbfgs, l2 penalty regularization strength C=1), Principal Component Analysis in sequence with a linear model (scikit-learn default parameters version 1.5.1 PCA 10 components), and a deep-learning contrastive learning model using the recently presented CEBRA toolbox (Schneider et al., 2023). CEBRA contrastive learning was used with a five-layer convolutional neural network (“offset10-model”, 32 hidden units for the first layer, followed by three convolutional *skip layers* with each 32 hidden units and a four-dimensional convolutional output layer). The *skip layers* resembled a bottleneck across the temporal filter dimension from 10 to 3 samples. We used Gaussian Error Linear Unit (GELU) activation function for each layer, and applied normalization to the output layer. We specified the “auto” temperature mode with a learning rate of 0.005, and used in addition to the CEBRA embedding a linear model decoder for classification or regression (linear regression / logistic regression scikit-learn default parameters version 1.5.1, as above). We estimated performances for individual and combined feature modalities (raw amplitude, line length, aperiodic parameters, Hjorth parameters, burst, sharpwave, fft, welch and all combined tested separately). In addition, we investigated model performances with and without the time of the day “hour” feature. We quantified learning curves for different randomly selected training data durations (4, 8, 16, 32, 64, 128, 256, 512, 1024, 2048, 4096, 8192, 16384 min). For classification, we selected an equal number of positive and negative class samples. We computed feature importances using the CatBoost Predictive Value Change and investigated the top 10 features.

### Conectomic symptom-network decoding using Cleartune

We computed optimal stimulation parameters using *cleartune* (Rajamani et al., 2024), based on leave-one-patient-out CatBoost neural decoding performance. The monopolar stimulation parameters included both stimulation amplitude and active contact. We optimized stimulation parameters within 500 iterations, aiming to maximize engagement of white-matter tracts associated with bradykinesia and tremor, while dyskinesia predictions were used to inversely adjust the target stimulation amplitude within a range of 2.5–3.5 mA. Values outside this target amplitude were penalized as described in the orignial Rajamani et al 2024 paper (Rajamani et al., 2024). *cleartune* tracts were selected with a Volume of Tissue Activated (VTA) threshold of 0.5 V/mm. We applied stimulation parameter optimization to downsampled classification model predictions using moving-averaged symptom estimates every 12 minutes. We z-score normalized predictions such that the bradykinesia-to-tremor ratio was scaled between zero and one, resulting in inverse-proportional computation of bradykinesia and tremor tract activations. As a proof of concept, we conducted parameter optimization for patient 1, computing the percentage of previously reported bradykinesia and tremor tract activations (Rajamani et al., 2024) for each downsampled prediction step. Finally, we correlated neural symptom predictions with the relative proportion of tracts activated for bradykinesia and tremor. For Dyskinesia, we correlated model predictions with the estimated stimulation amplitudes.

### Statistics

We computed all statistical tests using non-parametric Monte-Carlo sampled permutation tests with a significance value of α=0.05. We report correlation values using Spearman’s rho correlation coefficient.

## Supplementary Material

1

## Figures and Tables

**Table 1 –:** Patient characteristics. UPDRS Tremor ratings are derived from the contralateral UPDRS III items 15-17 in the OFF-medication state. The UPDRS OFF-ON score is the percentage change in overall motor symptoms (UPDRS-III) between medication OFF and ON states. The OFF medication state is defined as 12 h after withdrawal of antiparkinsonian medication, and the ON-medication state is defined as 30-45 min after a supratherapeutic dose of carbidopa-levodopa. GP- Globus Pallidus. STN – Subthalamic Nucleus; MOCA – Montreal Cognitive Assessment

Subject ID	Motor signs (yrs)	UPDRS III (OFF)	UPDRS IV.1	UPDRS tremor sum	UPDRS (OFF-ON)	Implant site	Implanted sides	Subject age (yrs)	Gender
1	7	49	4	2	90	STN	bilateral	54	m
2	19	45	1	7	51	STN	bilateral	63	m
3	12	61	2	21	73	STN	bilateral	28	f
4	4	41	1	9	65	STN	bilateral	40	m
5	12	44	2	0	75	STN	bilateral	58	m
6	10	39	2	20	59	GP	bilateral	48	m
7	13	89	0	20	53	GP	bilateral	64	f
8	7	29	2	4	58	STN	bilateral	71	m
9	10	34	2	14	73	STN	bilateral	58	m
10	4	31	0	11	51	GP	unilateral	65	m
11	6	35	0	14	67	STN	bilateral	61	m
12	12	32	1	6	84	STN	bilateral	45	m
13	7	49	2	2	76	STN	bilateral	67	m
14	8	49	0	17	61	GP	bilateral	51	m
15	13	31	1	8	67	STN	bilateral	73	m
16	9	66	1	23	64	GP	unilateral	66	m

**Table 2:** Machine leanring leave one hemisphere out cross-validation and subcortical beta performances. Performances are shown Pearson correlation coefficient.

Label	Location	Pearson corr. (r)
Bradykinesia	ML ECoG + DBS electrodes	**0.61 ± 0.20**
	ML ECoG electrode	0.54 ± 0.21
	ML DBS electrode	0.50 ± 0.23
	Beta DBS	0.29 ± 0.31
	Beta STN	0.24 ± 0.33
	Beta GP	0.48 ± 0.15
Tremor	ML ECoG + DBS electrodes	**0.36 ± 0.32**
	ML ECoG electrode	0.27 ± 0.27
	ML DBS electrode	0.28 ± 0.31
	Beta DBS	0.24 ± 0.40
	Beta STN	0.22 ± 0.38
	Beta GP	0.32 ± 0.45
Dyskinesia	ML ECoG + DBS electrodes	**0.45 ± 0.27**
	ML ECoG electrode	0.40 ± 0.25
	ML DBS electrode	0.43 ± 0.29
	Beta DBS	−0.30 ± 0.26
	Beta STN	−0.32 ± 0.26
	Beta GP	−0.21 ± 0.22

## Data Availability

Trained models using data from all included subjects separate for classification and regression, using separate cortical, subcortical and combined locations, and all reported symptoms scores are made publicly available on a GitHub repository including required code for feature computation and model training and inference: https://github.com/neuromodulation/PD-neural-symptom-decoding-inference. Required code for analysis and figure reproduction is made available on a separate GitHub repository: https://github.com/timonmerk/PD_symptom_decoding.

## Data availability

Neural and wearable data can be made available upon request.
