# Supplementary material for: Multitarget brain implants enable generalized decoding of Parkinson’s disease symptoms from chronic home recordings
Source: Res Sq. 2026 May 15:rs.3.rs-9125364. Preprint. [Version 1] doi: 10.21203/rs.3.rs-9125364/v1 (PMC13193127; doi:10.21203/rs.3.rs-9125364/v1)
Supplement: 1 [file NIHPPrs9125364v1-supplement-1.pdf]

Supplementary Figures

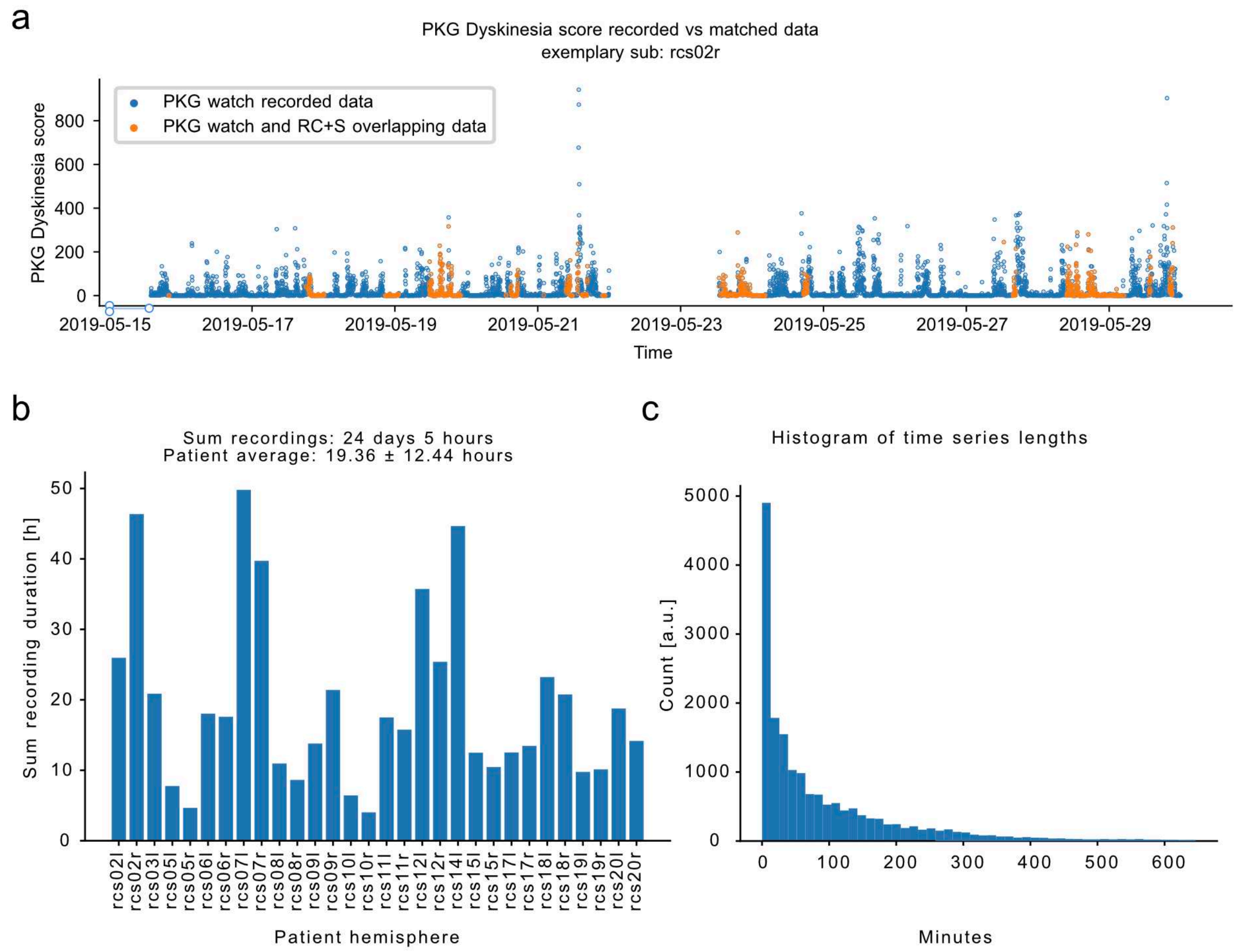

Supplementary Figure 1 – **Recording characteristics.** a) Invasive cortical and subcortical electrophysiology was recorded using the Summit RC+S neurostimulator. In addition, wearable symptoms were recorded using the Personal KinetiGraph (PKG) watch. For an exemplary patient (rcs02r) electrophysiological and wearable sample times are shown. During the recording duration, data was recorded non-continuously with intermitted breaks. b) Hemisphere-individual recording durations. The overall analyzed recording duration was 24 days and 5 hours. c) Histogram of consecutive time series lengths. The overall mean continuous time series length was 44.83 ± 56.77 min.

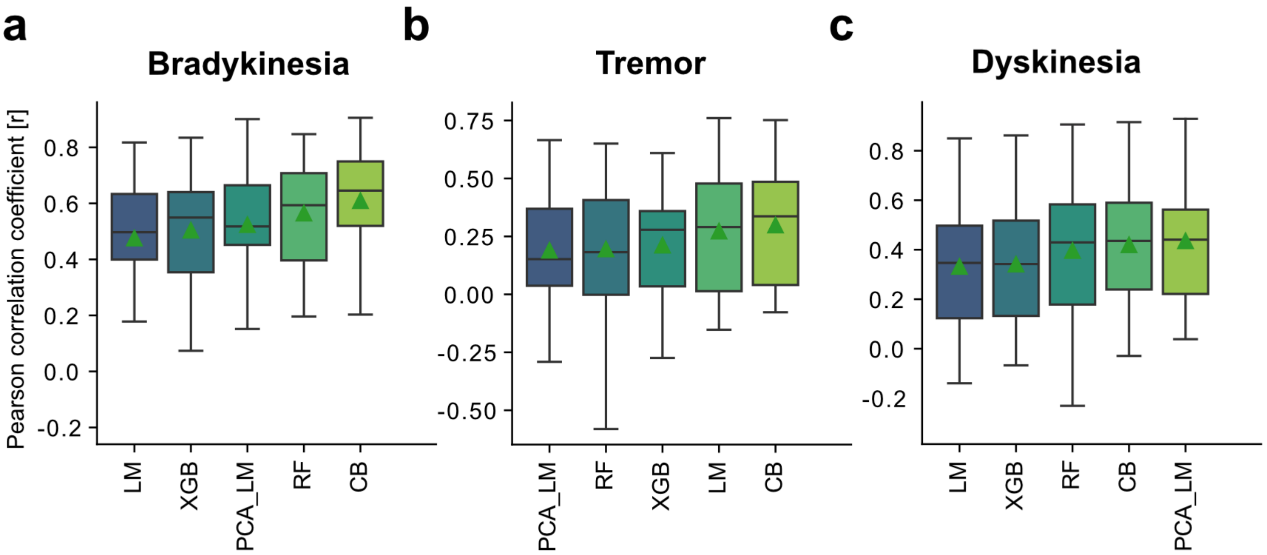

Supplementary Figure 2 – **Neural decoding performance for difference machine learning algorithms.** Performances are separated for different symptoms: a) bradykinesia, b) tremor and c) dyskinesia.

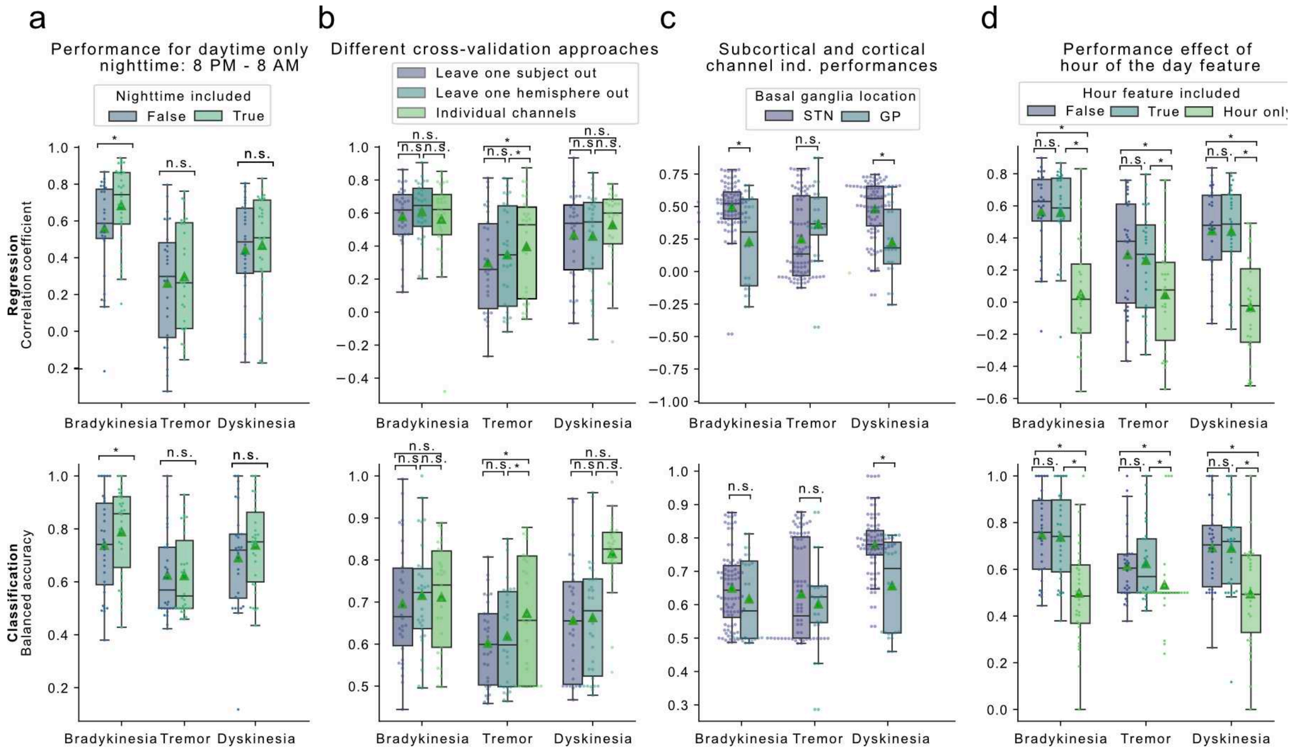

Supplementary Figure 3 - **Performance comparison of different recording times, sites and cross-validation strategies.** a) Performance distribution with and without excluded nighttime (8 PM- 8 AM). b) Performance comparison of different cross-validation strategies: leave one subject out, leave one hemisphere out and individual channel cross-validation. c) Performance analysis within channel-individual non-shuffled three-fold cross-validation with respect to different recording locations: Subcortical Subthalamic Nucleus (STN) and Globus Pallidus (GP). d) Performance comparison for neural features, or with time "hour of the day" feature and "hour of the day" feature alone.

Supplementary Video

Supplementary Video 1 – **Time-resolved symptom tract activation and stimulation profiles.** *cleartune* (Rajamani et al., 2024) was used to optimize stimulation contact parameters resulting in time-resolved tract activations with a sampling rate of 12 minutes using bradykinesia, tremor and dyskinesia decoding predictions.

Supplementary Files

This is a list of supplementary files associated with this preprint. Click to download.

- SupplementaryVideo1.mp4
